# The longitudinal effect of the aldehyde dehydrogenase 2*2 allele on the risk for nonalcoholic fatty liver disease

**DOI:** 10.1038/nutd.2016.17

**Published:** 2016-05-23

**Authors:** K Oniki, K Morita, T Watanabe, A Kajiwara, K Otake, K Nakagawa, Y Sasaki, Y Ogata, J Saruwatari

**Affiliations:** 1 Division of Pharmacology and Therapeutics, Graduate School of Pharmaceutical Sciences, Kumamoto University, Kumamoto, Japan; 2 Department of Gastroenterology and Hepatology, Faculty of Life Sciences, Kumamoto University, Kumamoto, Japan; 3 Japanese Red Cross Kumamoto Health Care Center, Kumamoto, Japan; 4 Center for Clinical Pharmaceutical Sciences, Kumamoto University, Kumamoto, Japan

## Abstract

Aldehyde dehydrogenase 2 (ALDH2) detoxifies toxic aldehydes and has a key role in protecting the liver. An elevated gamma-glutamyl transferase (GGT) level is related to oxidative stress and nonalcoholic fatty liver disease (NAFLD). We herein investigated the association between inactive ALDH2*2 allele (rs671) and the risk of NAFLD, including the relationship to the GGT level. A retrospective follow-up study (mean 5.4±1.1 years) was conducted among 341 Japanese health screening program participants. The receiver operating characteristic curve indicated that the GGT level predicted the development of NAFLD (area under the curve: 0.65, *P*<0.05) with a cutoff value of 25.5 IUl^−1^. The longitudinal risk of NAFLD was higher in the ALDH2*2 allele carriers than in the noncarriers (odds ratio (OR): 2.30, 95% confidence interval (CI): 1.21–4.40), and the risk was further increased among the *2 allele carriers with GGT values ⩾25.5 IUl^−1^ (OR: 4.28, 95% CI: 1.80–10.19). On the other hand, there were no significant changes in the subjects' body weight and body mass index during observation period. The ALDH2*2 allele, in relation to the GGT level, may potentially be a novel risk factor for NAFLD.

## Introduction

Nonalcoholic fatty liver disease (NAFLD) is the most frequent chronic liver disease, which is recognized as a liver manifestation of metabolic syndrome, and it is a significant predictor of future coronary artery disease.^1, 2, 3^ Oxidative stress is known to be of major importance in the development and progression of NAFLD.^1, 2, 3^ Aldehyde dehydrogenase 2 (ALDH2) expressed in the liver detoxifies toxic aldehydes, such as acetaldehyde, 4-hydroxynonenal and malondialdehyde (derived from alcohol and/or generated by lipid peroxidation), and has a key role in protection from oxidative injury.^4, 5^ The ALDH2*1 and *2 (rs671) alleles encode the active and inactive subunits of ALDH2, respectively, the latter of which determines an individual's tolerance for alcohol consumption.^4, 5^ Indeed, the active ALDH2*1/*1 genotype was identified to be a risk factor for alcoholism, alcohol-induced liver diseases and hypertension.^6, 7^ The inactive ALDH2*2 allele was identified to be a risk factor for coronary artery disease according to a meta-analysis of a genome-wide association study in East Asians.^8^ As Stachowicz *et al.*^9^ recently demonstrated that the activation of ALDH2 attenuated both atherosclerosis and NAFLD in apolipoprotein E-knockout mice, we hypothesized that the ALDH2*2 allele is associated with an increased risk for NAFLD, however, there is presently no data available regarding this association.

The gamma-glutamyl transferase (GGT) level is recognized as a marker of not only excessive alcohol consumption or liver injury, but also oxidative stress,^10^ and it is a significant predictor for metabolic syndrome and NAFLD, independent of alcohol consumption.^11, 12^ In our recent study, the combination of a mildly elevated GGT value and harboring the ALDH2*2 allele was interactively associated with the incidence of diabetic retinopathy.^13^

According to this information, the present exploratory study aimed to investigate whether the *ALDH2* rs671 polymorphism could affect the risk for NAFLD using a longitudinal association analysis, while also paying careful attention to the GGT level.

## Subjects and methods

A retrospective longitudinal analysis with 5.4±1.1 years of follow-up was performed consisting of 341 subjects who were consecutively recruited from health screening program participants in the Japanese Red Cross Kumamoto Health Care Center between January 2006 and April 2012. All subjects were non or moderate drinkers (consuming <30 g per day of alcohol in males and 20 g per day in females), hepatitis B and C virus-negative, and did not have autoimmune liver disease, hepatocellular carcinoma, primary biliary cirrhosis, Wilson's disease or drug-induced hepatitis. The study protocol was approved by the institutional ethics committees, and written informed consent was obtained from each patient.

The diagnosis of FLD was performed by hepatic ultrasonography scanning by a radiographer. A medical doctor then reviewed the images to evaluate the accuracy and reproducibility of the diagnosis. In addition, both radiographer and medical doctor performed the diagnosis of FLD, blinded to information regarding the clinical features of subjects. Genomic DNA was extracted from the whole blood using a DNA purification kit (FlexiGene DNA kit; Qiagen, Hilden, Germany). The *ALDH2* rs671 (*2) and *patatin-like phospholipase domain-containing 3* (*PNPLA3*) rs738409 (c.444C>G) polymorphisms were determined using a real-time TaqMan allelic discrimination assay (Applied Biosystems, Foster City, CA, USA) according to the manufacturer's protocols. In this study, the *PNPLA3* rs738409 polymorphism was investigated for the adjustment during the statistical analyses, because it is known to be the most frequently reported genetic predictor for NAFLD.^14^

Categorical valuables were compared using Fisher's exact test. Student's *t*-test or one-way analysis of variance and the Mann–Whitney *U*-test or Kruskal–Wallis test were used to compare the differences in the parametric and nonparametric valuables, respectively. A receiver operating characteristic (ROC) curve was determined to evaluate the predictive performance of the GGT values at baseline for detecting NAFLD during the follow-up period with calculations of the area under the curve, and the cutoff value of the GGT level as the point with the shortest distance from the left upper corner of the ROC curve was calculated. The longitudinal effects of the *ALDH2* genotype and the combination of the genotype, and a higher GGT level (i.e., greater than the cutoff value) on the risk for NAFLD were analyzed among all subjects and the nondrinkers (defined as one drink or less per month of alcohol intake) using a multivariable logistic regression model. In this model, the odds ratios and 95% confidence intervals (CIs) were measured according to the generalized estimating equations approach as we reported previously.^15^ The fitness of the multivariable logistic regression models was determined using the quasi-likelihood criterion. According to the final logistic regression models, the C-statistics, that is, area under the curve values, with 95% CI of the ROC curve were also calculated using the logit function of the individual probability values for the prevalence of NAFLD. Moreover, a stratified nonparametric bootstrap analysis was performed to investigate the precision of the parameters of the logistic regression model regarding the risk of NAFLD. Five thousand replicated data sets were generated by random sampling with replacement, and stratified according to the study population, to ensure a representative study population distribution using the individual as the sampling unit. A two-tailed *P*-value <0.05 was considered to be statistically significant. All statistical analyses were performed using the SPSS software package (version 23.0, IBM Japan Inc., Tokyo, Japan).

## Results

**Figure 1:**
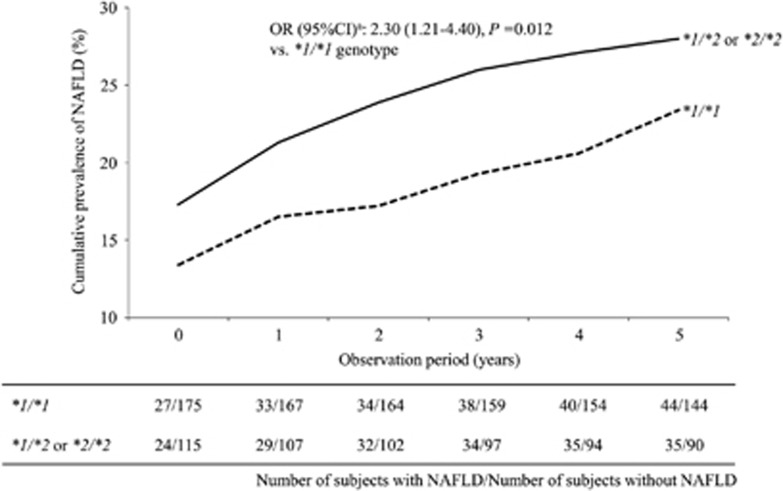
The prevalence of NAFLD according to the *ALDH2* genotypes during the follow-up period.

The *ALDH2* genotype frequency distribution was consistent with the Hardy–Weinberg equilibrium (*P*>0.05). The clinical characteristics of the subjects stratified by the ALDH2*2 allele and the genotype at baseline are shown in Supplementary Tables 1 and 2, respectively. According to the ROC curve, the GGT level at baseline was found to be a significant predictor of NAFLD (area under the curve: 0.65, 95% CI: 0.58–0.72, *P*<0.001), and the cutoff value was determined to be 25.5 IUl^−1^ (sensitivity: 58.4%, specificity: 67.8%). At baseline, the prevalence of NAFLD tended to be higher in the ALDH2*2 allele carriers than in the noncarriers, although the association did not reach statistical significance (Supplementary Table 1). The logistic regression models showed that the longitudinal risk of NAFLD was significantly higher in the ALDH2*2 allele carriers than in the noncarriers (Figure 1), and the risk was further increased among the *2 allele carriers with a GGT level of ⩾25.5 IUl^−1^ (Table 1). Among the 159 nondrinkers, the logistic regression models showed that the longitudinal risk of NAFLD also increased in the ALDH2*2 allele carriers (odds ratio: 4.52, 95% CI: 1.45–14.08) and in those with a GGT level of ⩾25.5 IUl^−1^ (odds ratio: 6.25, 95% CI: 1.58–24.69). The quasi-likelihood criterion values of the models with the *ALDH2* genotype, and with the combination of *ALDH2* genotype and GGT level, were 1493.4 and 1516.2, respectively, for all subjects; and 695.8 and 691.3, respectively, for nondrinkers, and were lower than those of base models (2083.6 for all subjects and 968.6 for nondrinkers). The C-statistics (95% CI) of the ROC curves regarding the models with the *ALDH2* genotype, and with the combination of *ALDH2* genotype and GGT level, were 0.869 (0.851–0.887) and 0.873 (0.856–0.891), respectively, for all subjects; and 0.878 (0.853–0.903) and 0.889 (0.866–0.912), respectively, for nondrinkers. These C-statistic values were statistically significant (*P*<0.001). Moreover, in the bootstrap analysis, the models using the 5000 replicated data sets also indicated that the ALDH2*2 allele and in combination with a GGT level of ⩾25.5 IUl^−1^ were associated with the risk of NAFLD (odds ratio (95% CI): 1.49 (1.29–1.72) and 4.38 (3.57–5.36), respectively, for all subjects; 2.21 (1.90–2.57) and 4.36 (3.63–5.24), respectively, for nondrinkers). Therefore, the present logistic regression models adequately described the original data, and the overall fitness of our final models were observed to be good. A statistical interaction between the ALDH2*2 allele and GGT level on the risk for NAFLD was not observed (*P*>0.05). There were no significant changes in the subjects' body weight, body mass index and GGT value during the observation period, including the stratified analyses by the ALDH2*2 allele (data not shown). In addition, the effects of the ALDH2*2/*2 genotype on the risk for NAFLD are shown in Supplementary Table 3, although the number of *2/*2 genotype carriers was small.

## Discussion

To the best of our knowledge, this is the first report to show that the ALDH2*2 allele is associated with a risk for NAFLD in relation to an elevated GGT level.

ALDH2 has a beneficial role in ameliorating chronic alcohol-induced hepatic steatosis and inflammation.^16^ Recent studies have shown that accelerated detoxification of toxic aldehydes through the activation of ALDH2 by Alda-1 attenuated NAFLD as well as alcohol-induced hepatic steatosis.^9, 17^ In the present study, we found significant influences of the ALDH2*2 allele on the risk for NAFLD, without any increases in the patient body weight among nondrinkers as well as all subjects. Although the amount of alcohol intake differs between *ALDH2* genotypes,^4, 5^ the *2 allele carriers may be a high-risk group for FLD due to increased toxic aldehydes derived from alcohol and/or other factors (e.g., unhealthy diet, low physical activity, aging and diseases) among light drinkers or nondrinkers.

We showed that an elevated GGT level at baseline predicted the development of NAFLD even within the normal range, especially in the ALDH2*2 allele carriers (Table 1). GGT metabolizes extracellular glutathione, allowing precursor amino acids to be reused for intracellular glutathione synthesis; hence, a modest increase within the normal range may be an early marker of oxidative stress.^10^ In the previous reports, the ALDH2*2 allele, in combination with a mild elevation in the GGT level, drinking habit and/or smoking habit, was associated with an increased risk for hypertension,^18^ diabetic retinopathy,^13^ myocardial infarction^19^ or chronic airway obstruction.^15^ According to these findings, ALDH2 may therefore have a critical role in the protection against toxic aldehydes generated under sustained high oxidative stress conditions, and our findings thus suggest that the GGT level should be carefully monitored for the prevalence of NAFLD, especially in ALDH2*2 allele carriers.

There are some limitations associated with the present study. Although the overall fits of the models were observed to be good, the present study had a small sample size and was a single-center retrospective observational study. Because the study subjects were health screening program participants, they might have had a high level of health literacy. In addition, we included Japanese health screening program participants only; therefore, there may be a possibility that a selection bias is associated with the results of the present study, and it is unclear whether the present results can be generalized to other populations. To verify the results observed in the present study, further multicenter prospective studies in larger and more diverse populations are required. The subjects' alcohol consumption was evaluated through face-to-face interviews, which may have lacked reliability. Because the study subjects were participants of a health screening program (i.e., relatively healthy population), the diagnosis of NAFLD could not be confirmed by a liver biopsy. Among the NAFLD subjects, the values of the Fibrosis-4 index (noninvasive index for liver fibrosis)^20^ and alanine aminotransferase were relatively low (Supplementary Table 4), and most of the NAFLD subjects were thought to have simple steatosis. The associations of the ALDH2*2 allele with the risk for NAFLD were not observed in the bi-variable models (Supplementary Table 5). There was a difference in the female frequency between *2 allele carriers and noncarriers (Supplementary Table 1), and thus the bi-variable model might not be appropriate.

In conclusion, this study provided the preliminary findings showing that the inactive ALDH2*2 allele may potentially be a novel risk factor for NAFLD in relation to an elevated GGT level. These findings may be utilized for the health promotion of the high-risk group for NAFLD (i.e., ALDH2*2 allele carriers with GGT ⩾25.5 IUl^−1^) and/or therapeutics for NAFLD (e.g., ALDH2 activation using Alda-1 for the high-risk group), although further investigations with a greater accumulation of subjects are needed before any definitive conclusions can be made.

## Figures and Tables

**Table 1:** The effect of the combination of the ALDH2*2 allele and the GGT level on the risk for NAFLD in a longitudinal multivariable logistic regression analysis

	*OR (95% CI)*^a^	P*-value*
*Combination of the ALDH2 genotype and the GGT level*		
*1/*1 genotype with GGT level <25.5 IUl^−1^	1	—
*1/*1 genotype with GGT level ⩾25.5 IUl^−1^	1.53 (0.68–3.45)	0.308
*1/*2 or *2/*2 genotype with GGT level <25.5 IUl^−1^	1.92 (0.82–4.50)	0.132
*1/*2 or *2/*2 genotype with GGT level ⩾25.5 IUl^−1^	4.28 (1.80–10.19)	0.001
		
*Gender*		
* *Male	1	—
* *Female	2.82 (1.47–5.42)	0.002
		
BMI (kgm^−2^)	1.49 (1.30–1.71)	<0.001
HDL-C (mgdl^−1^)	0.96 (0.94–0.99)	0.003
TG (mgdl^−1^)	1.01 (1.002–1.013)	0.007
		
*Diabetes*		
* *Absent	1	—
* *Present	2.61 (1.30–5.22)	0.007
		
*PNPLA3*		
* C/C* genotype	1	—
* C/G* or *G/G* genotype	3.42 (1.59–7.33)	0.002

Abbreviations: ALDH2, aldehyde dehydrogenase 2; BMI, body mass index; CI, confidence interval; GGT, gamma-glutamyl transferase; HDL-C, high-density lipoprotein cholesterol; NAFLD, nonalcoholic fatty liver disease; OR, odds ratio; *PNPLA3*, patatin-like phospholipase domain-containing 3; TG, triglycerides.

a Adjusted by all covariates.
