# Supplementary material for: The longitudinal effect of the aldehyde dehydrogenase 2*2 allele on the risk for nonalcoholic fatty liver disease
Source: Nutr Diabetes. 2016 May 23;6(5):e210–. doi: 10.1038/nutd.2016.17 (PMC4895378; doi:10.1038/nutd.2016.17)
Supplement: Supplementary Table 1 [file nutd201617x1.docx]

Supplemental Table 1. Clinical characteristics at baseline of the subjects stratified by harboring the *ALDH2*2* allele.

|  | | All subjects  (n = 341) |  | **1/*1*  (n = 202) | **1/*2* or **2/*2*  (n = 139) | *P* value |
| --- | --- | --- | --- | --- | --- | --- |
| Female (%) | | 145 (42.5) |  | 95 (47.0) | 50 (36.0) | 0.045 ^a^ |
| Age (years) | | 67.7 ± 5.9 |  | 67.7 ± 5.9 | 67.6 ± 5.8 | 0.875 |
| BMI (kg/m^2^) | | 22.6 ± 2.8 |  | 22.8 ± 2.8 | 22.3 ± 2.8 | 0.087 |
| Waist circumstance (cm) | | 82.6 ± 7.8 |  | 82.8 ± 7.8 | 82.3 ± 7.8 | 0.630 |
| Fasting blood glucose (mg/dL) | | 97 (91 - 106) |  | 96 (91 - 107) | 97 (91 - 105) | 0.894 ^b^ |
| Systolic BP (mmHg) | | 122.8 ± 17.6 |  | 123.8 ± 17.4 | 121.2 ± 17.9 | 0.191 |
| Diastolic BP (mmHg) | | 71.9 ± 10.7 |  | 72.8 ± 10.7 | 70.6 ± 10.7 | 0.065 |
| LDL-C (mg/dL) | | 125.5 ± 26.7 |  | 125.4 ± 25.8 | 125.6 ± 27.9 | 0.950 |
| HDL-C (mg/dL) | | 69.5 ± 16.6 |  | 70.1 ± 17.0 | 68.5 ± 16.1 | 0.366 |
| TG (mg/dL) | | 91 (68 - 123) |  | 92 (68 - 123) | 90 (69 - 123) | 0.708 ^b^ |
| AST (IU/L) | | 23.9 ± 6.7 |  | 24.0 ± 6.9 | 23.8 ± 6.6 | 0.805 |
| ALT (IU/L) | | 21.6 ± 9.6 |  | 22.0 ± 10.6 | 21.1 ± 7.9 | 0.379 |
| GGT (IU/L) | | 22 (16 - 32) |  | 22 (16 - 33) | 23 (17 - 32) | 0.382 ^b^ |
| Diabetes (%) | | 43 (12.6) |  | 29 (14.4) | 14 (10.1) | 0.319 ^a^ |
| Hypertension (%) | | 140 (41.1) |  | 87 (43.1) | 53 (38.1) | 0.373 ^a^ |
| Dyslipidemia (%) | | 164 (48.1) |  | 94 (46.5) | 70 (50.4) | 0.509 ^a^ |
| NAFLD (%) | | 51 (15.0) |  | 27 (13.4) | 24 (17.3) | 0.355 ^a^ |
| Ever-smokers (%) | | 114 (33.4) |  | 61 (30.2) | 53 (38.1) | 0.131 ^a^ |
| *PNPLA3* | C/C (%) | 95 (27.9) |  | 53 (26.2) | 42 (30.2) | 0.574 ^a^ |
|  | C/G (%) | 187 (54.8) |  | 111 (55.0) | 76 (54.7) |  |
|  | G/G (%) | 59 (17.3) |  | 38 (18.8) | 21 (15.1) |  |

The data are the means ± standard deviation, median (interquartile range) for skewed variables, or the number of the subjects (%) for categorical variables.

^a^ Fisher’s exact test. ^b^ Mann-Whitney U test (otherwise, Student’s t-test was used).

AST, aspartate aminotransferase; ALT, alanine aminotransferase; BMI, body mass index; BP, blood pressure; GGT, gamma-glutamyl transferase; HDL-C, high-density lipoprotein cholesterol; LDL-C, low-density lipoprotein cholesterol; NAFLD, non-alcoholic fatty liver disease; PNPLA3, patatin-like phospholipase 3; TG, triglyceride.
