# Supplementary material for: The longitudinal effect of the aldehyde dehydrogenase 2*2 allele on the risk for nonalcoholic fatty liver disease
Source: Nutr Diabetes. 2016 May 23;6(5):e210–. doi: 10.1038/nutd.2016.17 (PMC4895378; doi:10.1038/nutd.2016.17)
Supplement: Supplementary Table 2 [file nutd201617x2.docx]

Supplemental Table 2. Clinical characteristics at baseline of the subjects stratified by the *ALDH2* genotype.

|  | | **1/*1*  (n = 202) | **1/*2*  (n = 118) | **2/*2*  (n = 21) | *P* value |
| --- | --- | --- | --- | --- | --- |
| Female (%) | | 95 (47.0) | 42 (35.6) | 8 (38.1) | 0.120 ^a^ |
| Age (years) | | 67.7 ± 5.9 | 67.4 ± 5.8 | 68.9 ± 5.6 | 0.565 |
| BMI (kg/m^2^) | | 22.8 ± 2.8 | 22.6 ± 2.6 | 20.6 ± 3.4 | 0.003 |
| Waist circumstance (cm) | | 82.8 ± 7.8 | 83.1 ± 7.7 | 78.2 ± 7.3 | 0.061 |
| Fasting blood glucose (mg/dL) | | 96 (91 - 107) | 97 (91 - 105) | 98 (88 - 103) | 0.977 ^b^ |
| Systolic BP (mmHg) | | 123.8 ± 17.4 | 122.1 ± 17.9 | 116.2 ± 17.3 | 0.154 |
| Diastolic BP (mmHg) | | 72.8 ± 10.7 | 70.8 ± 11.0 | 69.4 ± 9.5 | 0.157 |
| LDL-C (mg/dL) | | 125.4 ± 25.8 | 125.2 ± 29.2 | 127.8 ± 19.8 | 0.917 |
| HDL-C (mg/dL) | | 70.1 ± 17.0 | 67.9 ± 16.2 | 72.0 ± 15.0 | 0.383 |
| TG (mg/dL) | | 92 (68 - 123) | 93 (70 - 127) | 74 (57 - 115) | 0.150 ^b^ |
| AST (IU/L) | | 24.0 ± 6.9 | 23.8 ± 6.2 | 23.8 ± 8.6 | 0.970 |
| ALT (IU/L) | | 22.0 ± 10.6 | 21.3 ± 7.7 | 20.1 ± 8.9 | 0.588 |
| GGT (IU/L) | | 22 (16 - 33) | 23 (17 - 32) | 22 (15 - 38) | 0.534 ^b^ |
| Diabetes (%) | | 29 (14.4) | 13 (11.0) | 1 (4.8) | 0.437 ^a^ |
| Hypertension (%) | | 87 (43.1) | 47 (39.8) | 6 (28.6) | 0.427 ^a^ |
| Dyslipidemia (%) | | 94 (46.5) | 61 (51.7) | 9 (42.9) | 0.581 ^a^ |
| NAFLD (%) | | 27 (13.4) | 23 (19.5) | 1 (4.8) | 0.157 ^a^ |
| Ever-smokers (%) | | 61 (30.2) | 46 (39.0) | 7 (33.3) | 0.263 ^a^ |
| *PNPLA3* | C/C (%) | 53 (26.2) | 34 (28.8) | 8 (38.1) | 0.760 ^a^ |
|  | C/G (%) | 111 (55.0) | 66 (55.9) | 10 (47.6) |  |
|  | G/G (%) | 38 (18.8) | 18 (15.3) | 3 (14.3) |  |

The data are the means ± standard deviation, median (interquartile range) for skewed variables, or the number of the subjects (%) for categorical variables.

^a^ Fisher’s exact test. ^b^ Kruskal-Wallis test (otherwise, one-way analysis of variance).

AST, aspartate aminotransferase; ALT, alanine aminotransferase; BMI, body mass index; BP, blood pressure; GGT, gamma-glutamyl transferase; HDL-C, high-density lipoprotein cholesterol; LDL-C, low-density lipoprotein cholesterol; NAFLD, non-alcoholic fatty liver disease; PNPLA3, patatin-like phospholipase 3; TG, triglyceride.
