# Supplementary material for: The longitudinal effect of the aldehyde dehydrogenase 2*2 allele on the risk for nonalcoholic fatty liver disease
Source: Nutr Diabetes. 2016 May 23;6(5):e210–. doi: 10.1038/nutd.2016.17 (PMC4895378; doi:10.1038/nutd.2016.17)
Supplement: Supplementary Table 3 [file nutd201617x3.docx]

Supplemental Table 3. The effect of the *ALDH2* genotype on the risk for NAFLD in a longitudinal multivariable logistic regression analysis.

|  | OR (95% CI) ^a^ | *P* value |
| --- | --- | --- |
| **1/*1* genotype | 1 | - |
| **1/*2* genotype | 2.28 (1.17 - 4.44) | 0.015 |
| **2/*2* genotype | 1.48 (0.35 - 6.32) | 0.594 |
|  |  |  |
| Combination of the *ALDH2* genotype and the GGT level |  |  |
| **1/*1* genotype with GGT level <25.5 IU/L | 1 | - |
| **1/*1* genotype with GGT level ≥25.5 IU/L | 1.81 (0.79 - 4.15) | 0.159 |
| **1/*2* genotype with GGT level <25.5 IU/L | 1.82 (0.74 - 4.48) | 0.190 |
| **1/*2* genotype with GGT level ≥25.5 IU/L | 5.03 (2.07 - 12.18) | < 0.001 |
| **2/*2* genotype with GGT level <25.5 IU/L | 2.96 (0.70 - 12.54) | 0.132 |
| **2/*2* genotype with GGT level ≥25.5 IU/L ^b^ | - | - |

^a^ Adjusted by gender, BMI, HDL-C, TG and *PNPLA3* genotype.

^b^ Data was not available.

ALDH2, aldehyde dehydrogenase 2; BMI, body mass index; CI, confidence interval; GGT, gamma-glutamyltransferase; HDL-C, high-density lipoprotein cholesterol; NAFLD, non-alcoholic fatty liver disease; OR, odds ratio; PNPLA3, patatin-like phospholipase domain-containing 3; TG, triglycerides.
