# Supplementary material for: The longitudinal effect of the aldehyde dehydrogenase 2*2 allele on the risk for nonalcoholic fatty liver disease
Source: Nutr Diabetes. 2016 May 23;6(5):e210–. doi: 10.1038/nutd.2016.17 (PMC4895378; doi:10.1038/nutd.2016.17)
Supplement: Supplementary Table 4 [file nutd201617x4.docx]

Supplemental Table 4. Clinical characteristics of the subjects at the end point of the follow-up period.

|  | | Without NAFLD  (n = 264) | With NAFLD  (n = 77) | *P* value |
| --- | --- | --- | --- | --- |
| Female (%) | | 117 (44.3) | 28 (36.4) | 0.240 ^a^ |
| Age (years) | | 73.4 ± 6.0 | 72.2 ± 5.7 | 0.143 |
| BMI (kg/m^2^) | | 21.8 ± 2.6 | 24.9 ± 2.9 | <0.001 |
| Waist circumstance (cm) | | 81.0 ± 7.9 | 88.9 ± 7.2 | <0.001 |
| Fasting blood glucose (mg/dL) | | 97 (91 - 105) | 104 (97 - 119) | <0.001 ^b^ |
| Systolic BP (mmHg) | | 123.6 ± 16.0 | 127.6 ± 15.0 | 0.053 |
| Diastolic BP (mmHg) | | 70.4 ± 9.6 | 69.0 ± 9.0 | 0.226 |
| LDL-C (mg/dL) | | 118.8 ± 24.6 | 125.1 ± 26.2 | 0.051 |
| HDL-C (mg/dL) | | 73.6 ± 16.8 | 58.5 ± 14.0 | <0.001 |
| TG (mg/dL) | | 81 (63 - 105) | 107 (89 - 139) | <0.001 ^b^ |
| AST (IU/L) | | 22.5 ± 6.0 | 23.8 ± 7.1 | 0.118 |
| ALT (IU/L) | | 18.3 ± 7.3 | 23.3 ± 11.3 | <0.001 |
| GGT (IU/L) | | 20 (15 - 28) | 25 (20 - 35) | <0.001 ^b^ |
| FIB4 index | | - | 1.81 (1.49 - 2.39) | - |
| FIB4 index ≥ 2.67 (%) | | - | 13 (16.9) | - |
| Diabetes (%) | | 40 (15.2) | 26 (33.8) | 0.001 ^a^ |
| Hypertension (%) | | 135 (51.1) | 42 (54.5) | 0.607 ^a^ |
| Dyslipidemia (%) | | 138 (52.3) | 55 (71.4) | 0.004 ^a^ |
| Ever-smokers (%) | | 92 (34.8) | 28 (36.4) | 0.892 ^a^ |
| *ALDH2* | **1/*1* (%) | 159 (60.2) | 43 (55.8) | 0.418 ^a^ |
|  | **1/*2* (%) | 87 (33.0) | 31 (40.3) |  |
|  | **2/*2* (%) | 18 (6.8) | 3 (3.9) |  |
| *PNPLA3* | C/C (%) | 80 (30.3) | 15 (19.5) | 0.099 ^a^ |
|  | C/G (%) | 143 (54.2) | 44 (57.1) |  |
|  | G/G (%) | 41 (15.5) | 18 (23.4) |  |

The data are the means ± standard deviation, median (interquartile range) for skewed variables, or the number of the subjects (%) for categorical variables.

^a^ Fisher’s exact test. ^b^ Mann-Whitney U test (otherwise, Student’s t-test was used).

AST, aspartate aminotransferase; ALT, alanine aminotransferase; BMI, body mass index; BP, blood pressure; GGT, gamma-glutamyl transferase; HDL-C, high-density lipoprotein cholesterol; LDL-C, low-density lipoprotein cholesterol; NAFLD, non-alcoholic fatty liver disease; PNPLA3, patatin-like phospholipase 3; TG, triglyceride.
