# Supplementary material for: The longitudinal effect of the aldehyde dehydrogenase 2*2 allele on the risk for nonalcoholic fatty liver disease
Source: Nutr Diabetes. 2016 May 23;6(5):e210–. doi: 10.1038/nutd.2016.17 (PMC4895378; doi:10.1038/nutd.2016.17)
Supplement: Supplementary Table 5 [file nutd201617x5.docx]

Supplemental Table 5. The effect of the *ALDH2*2* allele on the risk for NAFLD in a longitudinal bi-variable logistic regression analysis.

|  | Non-adjusted  OR (95% CI) | *P* value |
| --- | --- | --- |
| **1/*1* genotype | 1 | - |
| **1/*2* or **2/*2* genotype | 1.33 (0.80 - 2.23) | 0.273 |
|  |  |  |
| Combination of the *ALDH2* genotype and the GGT level |  |  |
| **1/*1* genotype with GGT level <25.5 IU/L | 1 | - |
| **1/*1* genotype with GGT level ≥25.5 IU/L | 3.17 (1.56 - 6.42) | 0.001 |
| **1/*2* or **2/*2* genotype with GGT level <25.5 IU/L | 1.44 (0.66 - 3.12) | 0.357 |
| **1/*2* or **2/*2* genotype with GGT level ≥25.5 IU/L | 3.90 (1.84 - 8.25) | < 0.001 |

ALDH2, aldehyde dehydrogenase 2; CI, confidence interval; GGT, gamma-glutamyltransferase; NAFLD, non-alcoholic fatty liver disease; OR, odds ratio.
